# A Validated Model of the Pro- and Anti-Inflammatory Cytokine Balancing Act in Articular Cartilage Lesion Formation

**DOI:** 10.3389/fbioe.2015.00025

**Published:** 2015-03-10

**Authors:** Xiayi Wang, Marc J. Brouillette, Bruce P. Ayati, James A. Martin

**Affiliations:** ^1^Program in Applied Mathematical and Computational Sciences, University of Iowa, Iowa City, IA, USA; ^2^Department of Orthopaedics and Rehabilitation, University of Iowa, Iowa City, IA, USA; ^3^Department of Biomedical Engineering, University of Iowa, Iowa City, IA, USA; ^4^Department of Mathematics, University of Iowa, Iowa City, IA, USA

**Keywords:** articular cartilage, structured model, lesion formation and abatement, EPO, IL-6

## Abstract

Traumatic injuries of articular cartilage result in the formation of a cartilage lesion and contribute to cartilage degeneration and the risk of osteoarthritis (OA). A better understanding of the framework for the formation of a cartilage lesion formation would be helpful in therapy development. Toward this end, we present an age and space-structured model of articular cartilage lesion formation after a single blunt impact. This model modifies the reaction-diffusion-delay models in Graham et al. (2012) (single impact) and Wang et al. (2014) (cyclic loading), focusing on the “balancing act” between pro- and anti-inflammatory cytokines. Age structure is introduced to replace the delay terms for cell transitions used in these earlier models; we find age structured models to be more flexible in representing the underlying biological system and more tractable computationally. Numerical results show a successful capture of chondrocyte behavior and chemical activities associated with the cartilage lesion after the initial injury; experimental validation of our computational results is presented. We anticipate that our *in silico* model of cartilage damage from a single blunt impact can be used to provide information that may not be easily obtained through in *in vivo* or *in vitro* studies.

## 1 Introduction

The degenerative joint disease known as osteoarthritis (OA) is among the most common causes of disability worldwide. While OA involves multiple joint tissues including bone, tendons, ligaments and synovium, articular cartilage degeneration, and erosion is the proximal cause of loss of joint function. Articular cartilage is a thin layer of connective tissue that covers the ends of long bones in synovial joints such as the shoulder, hip, knee, and ankle, where it distribute mechanical loads and allows for smooth joint motion. These functions are attributable to the unique composition and structure of cartilage extracellular matrix (ECM), which consists of water (>70%), proteoglycan (15%), and collagen (15%) (Martin et al., 2011). Aggrecan, the major cartilage proteoglycan, is heavily decorated with negatively charged sulfate groups, which retain water in the tissue. Large complexes of aggrecan are trapped within the matrix by a collagen fibril network. The resistance of cartilage to compression and its ability to distribute loads is largely due to this macromolecular arrangement, and aggrecan depletion or collagen degradation radically reduces mechanical strength (Farndale et al., 1982; Lu et al., 2011).

Even though articular cartilage is only 1–3 mm thick, it has four distinct zones (superficial, transitional, radial, calcified). Different zones have different cell morphologies, matrix composition, and collagen fibril properties. Compared to other zones, the superficial zone is specialized to resist tensile stresses and minimize surface friction. In this paper, we focus on the properties of the superficial zone. We assume the solid cartilage matrix to be a homogeneous system, so that the chemical and cell properties remain the same inside the space. Cartilage cells, known as chondrocytes, are distributed sparsely within the tissue (104 cells/mm^3^). They are largely trapped inside the ECM, so there is no appreciable cell motility. Chondrocytes are solely responsible for the maintenance of the cartilage matrix, and engage in complex biochemical signaling/regulation via the synthesis and release or recognition of signaling molecules such as cytokines and oxidants, among others. We classify chondrocytes to be in different states in our model with respect to the chemical signaling processes. In addition to synthesizing ECM components, chondrocytes also release matrix proteases that cause matrix degradation.

**Figure 1:**
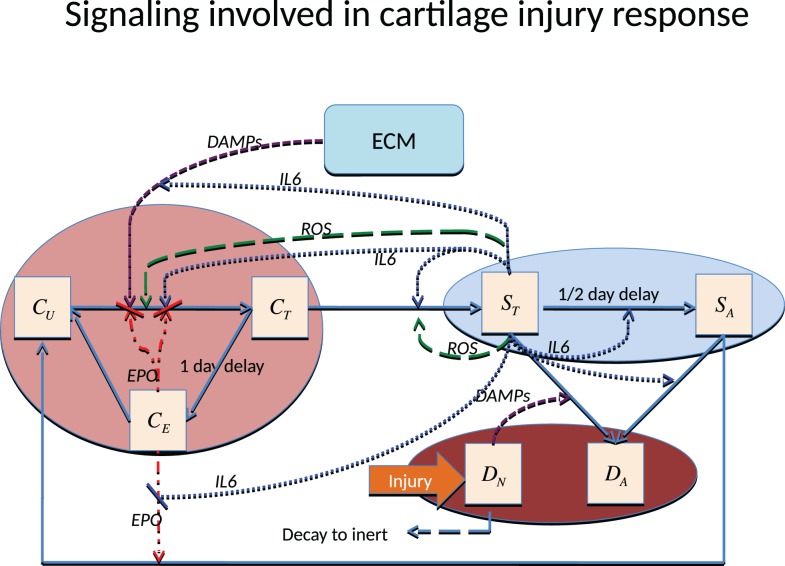
**Schematic of the articular cartilage lesion formation process due to a single blunt impact**.

There are various ways to cause traumatic articular cartilage injury, but they all share high loading rates and a high peak stress amplitude, which initiates the damage (lesion). The damage done by injuries seldom heals spontaneously and often leads to the progressive cartilage degeneration characteristic of post-traumatic osteoarthritis (PTOA). The strain in the superficial zone under a single blunt impact can easily exceed 40%, and when combined with the high loading rate and excessive stress, is lethal to chondrocytes and detrimental to the ECM. Chondrocyte death inside the superficial zone can be assumed to be directly caused by this impact. The main focus of most therapies for PTOA has been to prevent chondrocyte death and dysregulation. The chondrocyte depletion and ECM degradation process is illustrated in Figure 1. Cartilage damage initiates the production of alarmins, such as damage associated molecular patterns (DAMPs) (Bianchi, 2007), which can induce the release of pro-inflammatory cytokines such as interleukin-6 (IL-6), TNF-α, IL-1 α, and IL-1 β. A previous microarray study suggests that TNF-α might be the early mediator; however, IL-1 β is the cytokine that sustains the degradation (Martin et al., 2011). In this model, we use IL-6 to represent the entire cytokine family for model simplicity. Other cytokines such as TNF-α or IL-1 β can be easily added to the equations, but doing so would provide limited benefit with the need for additional parameter estimation and model complexity.

Pro-inflammatory cytokines are the main reason for cell apoptosis. Moreover, the pro-inflammatory cytokines can cause severe aggrecan depletion, which leads to loss of strength and elevated strain in affected cartilage. Even though it is quite limited, chondrocytes still have some self-repair ability. Anti-inflammatory cytokines such as erythropoietin (EPO) can antagonize the effect of pro-inflammatory cytokines and result in reduced cell apoptosis and ECM degradation. The “balancing act” (Graham et al., 2012) between pro-inflammatory and anti-inflammatory cytokines is the essential mechanism determining whether a cartilage lesion will heal or expand in size.

This paper is organized as follows. We describe the mathematical models and numerical methods used to solve the model equations. We then describe the materials and methods used for the experimental validation of our computational results. We then present the computational results and the experimental validation. We finish with a discussion of these results.

## 2 Materials and Methods

### 2.1 Mathematical model and numerical methods

In this section, we describe the age- and space-structured model developed for the inflammatory response after a single blunt impact injury. The cartilage lesion caused by a single severe traumatic event was described in a reaction-diffusion-delay model by Graham et al. (2012) and Graham (2013). In our model, we use age structure instead of delay terms to model the delayed transitions between cell states. This change in modeling approach was necessitated by the increased complexity of the interactions we are representing mathematically. Age structure is more efficient computationally than the use of delay terms, and is more flexible in representing transitions between cell states.

#### 2.1.1 Components of the system

We assume circular symmetry so that the system can be reduced to a one-dimensional model with respect to space. The components of the system depend on radius (*r*), age (*a*), and time (*t*). The time scales for our cell transitions are denoted by *τ*_1_ and *τ*_2_. We also assume that the initial blunt impact occurred on a small region near the origin with radius smaller than 0.25 cm, in a spatial domain with a total radius of 2.5 cm.

There are two main categories of components in our mathematical model, cells and chemicals. A schematic of the system is presented in Figure 1.

Let *r* denote radius, *a* time since signaled (i.e., age in current state), and *t* time (i.e., wall clock). The cell components are

- *C_U_* (*r*, *t*) = population density (cells per unit area) of healthy cells not yet signaled by ROS.
- *C_T_* (*r*, *a*, *t*) = population density of healthy cells signaled by ROS and in the process of becoming catabolic.
- *C_E_* (*r*, *t*) = population density of healthy cells signaled by ROS and starting to produce EPO (roughly 20–24 h after being signaled).
- *S_T_* (*r*, *a*, *t*) = population density of sick cells in the catabolic state. Healthy cells signaled by alarmins (DAMPs) and inflammatory cytokines (IL-6, TNF-α) enter into the catabolic state. Catabolic cells start to synthesize inflammatory cytokines and ROS.
- *S_A_* (*r*, *t*) = population density of EPOR-active cells. Catabolic cells signaled by inflammatory cytokines enter the EPOR-active state. EPOR-active cells express a receptor (EPOR) for EPO; however, there is a 8–12 h time gap before a cell expresses the EPO receptor after it was signaled to become EPOR-active (Brines and Cerami, 2008). After EPOR-active cells express a receptor for EPO, they may switch back to the healthy state *C_U_* when signaled by EPO.
- *D_A_* (*r*, *t*) = population density of apoptotic cells. Catabolic cells signaled by alarmins and inflammatory cytokines enter the apoptotic state. EPOR-active cells will also become apoptotic after signaled by inflammatory cytokines. Apoptotic cells do not play an explicit role in the mathematical model.
- *D_N_* (*r*, *t*) = population density of necrotic cells. In our model, necrotic cells are only created by the initial blunt impact and release alarmins (DAMPs) into the system. Before cells become necrotic, cells release a small amount of ROS. Fully necrotic cells cannot produce ROS and are eventually cleared from the system.

We assume negligible motility on the part of chondrocytes, so there are no diffusion terms for the cells equations. The different cell states correspond to the different chemical signals. The injury kills cells inside the impact area by rendering them necrotic (*D_N_*). The other cells adjacent to the impact area transit from “healthy” (*C_U_*, *C_T_*, *C_E_*) to “sick” (*S_T_*, *S_A_*) and then “dead” (apoptotic *D_A_*) states. Since apoptotic cells (*D_A_*) do not play an explicit role in the system, they are not expressed explicitly in our mathematical model, but are instead represented by the sink terms inside the *S_T_* and *S_A_* equations.

The chemical components are

- *R* (*r*, *t*) = concentration of reactive oxygen species (ROS). ROS triggers healthy cells to change states.
- *M* (*r*, *t*) = concentration of alarmins (DAMPs) released by necrotic cells and ECM degradation. DAMPs signal healthy cells *C_T_* to enter the catabolic state *S_T_*, and together with inflammatory cytokines such as IL-6, cause catabolic cells *S_T_* to become apoptotic.
- *F* (*r*, *t*) = concentration of inflammatory cytokines (IL-6) produced by catabolic cells (*S_T_*). These inflammatory cytokines
  – signals healthy cells (*C_T_*) to enter the catabolic state (*S_T_*),
  – signal catabolic cells (*S_T_*) to enter the EPOR-active state (Brines and Cerami, 2008),
  – cause both catabolic and EPOR-active cells to become apoptotic,
  – degrade the extracellular matrix, which in turn increases the level of DAMPs, resulting in further damage of the system. The degradation of ECM is a slow and complex process. However, we assume for mathematical convenience that inflammatory cytokines directly damage ECM,
  – limit the production of EPO.
- *P* (*r*, *t*) = concentration of erythropoietin (EPO). EPO is produced exclusively by *C_E_* in our model. Inflammatory cytokines suppress this process. EPO helps EPOR-active cells (*S_A_*) switch back to the healthy state *C_U_*. The effects of EPO depend on its concentration. When the concentration of EPO passes the threshold *P_c_* (Brines and Cerami, 2008), the spread of inflammation can be slowed by terminating the effect of inflammatory cytokines and alarmins on the system. We also assume that *C_E_* cells revert to *C_U_* when the EPO level exceeds the *P_c_* threshold.

We assume that the chemicals diffuse throughout the whole region. The diffusion coefficients were estimated by Graham et al. (2012) and Leddy and Guilak (2003). Chemicals decay after a certain time, and the decay rate can be approximated by their half lives (Eckardt et al., 1989; Wedlock et al., 1996; Ito et al., 2008). However, the decay of ROS is almost instantaneous under the superoxide dismute SOD, so the decay rate needs to be adjusted to fit the mathematical model. The inflammatory cytokines such as IL-6 and TNF-α are the main cause of cartilage lesion formation. EPO plays an opposing role by helping cell recovery and limiting the inflammation (Brines and Cerami, 2008). Our model captures the balance between these pro-inflammatory and anti-inflammatory cytokines.

In addition to the chemicals, we track the extracellular matrix density:

- *U*(*r*, *t*) = density of extracellular matrix (ECM). ECM is degraded by inflammatory cytokines and releases alarmins. The degradation of ECM is measured by the decrease in the concentration of SO_4_ (Farndale et al., 1982).

#### 2.1.2 Model equations

The equations for the chemical components of our system are

(1a) $$\begin{array}{ll} \partial_{t}\underset{\mathrm{ROS}}{\underset{︸}{R(r,t)}} & =\underset{\mathrm{diffusion}}{\underset{︸}{\frac{1}{r}\partial_{r}\left(rD_{R}R_{r}\right)}}-\underset{\mathrm{natural}\;\mathrm{decay}}{\underset{︸}{\delta_{R}R}}+\underset{\mathrm{production}\;\mathrm{by}\;S_{T}}{\underset{︸}{\sigma_{R}S_{T}}}, \end{array}$$

(1b) $$\begin{array}{ll} \partial_{t}\underset{\mathrm{DAMPs}}{\underset{︸}{M\left(r,t\right)}} & =\underset{\mathrm{diffusion}}{\underset{︸}{\frac{1}{r}\partial_{r}\left(rD_{M}M_{r}\right)}}-\underset{\mathrm{natural}\;\mathrm{decay}}{\underset{︸}{\delta_{M}M}}+\underset{\mathrm{production}\;\mathrm{by}\;D_{N}}{\underset{︸}{\sigma_{M}D_{N}}}+\underset{\mathrm{production}\;\mathrm{by}\;\mathrm{ECM}}{\underset{︸}{\delta_{U}U\frac{F}{\lambda_{F}+F}}}, \end{array}$$

(1c) $$\begin{array}{ll} \partial_{t}\underset{\mathrm{IL}-6}{\underset{︸}{F\left(r,t\right)}} & =\underset{\mathrm{diffusion}}{\underset{︸}{\frac{1}{r}\partial_{r}\left(rD_{F}F_{r}\right)}}-\underset{\mathrm{natural}\;\mathrm{decay}}{\underset{︸}{\delta_{F}F}}+\underset{\mathrm{production}\;\mathrm{by}\;S_{T}}{\underset{︸}{\sigma_{F}S_{T}}}, \end{array}$$

(1d) $$\begin{array}{l} \partial_{t}\underset{\mathrm{EPO}}{\underset{︸}{P\left(r,t\right)}}=\underset{\mathrm{diffusion}}{\underset{︸}{\frac{1}{r}\partial_{r}\left(rD_{P}P_{r}\right)}}-\underset{\mathrm{natural}\;\mathrm{decay}}{\underset{︸}{\delta_{P}P}}+\underset{\mathrm{production}\;\mathrm{by}\;C_{E},\;\mathrm{controlled}\;\mathrm{by}\;F}{\underset{︸}{\sigma_{P}C_{E}\frac{R}{\lambda_{R}+R}\frac{\Lambda }{\Lambda +F}}}. \end{array}$$

The initial and boundary conditions are

$$\partial_{r}R\left(0,t\right)=\partial_{r}M\left(0,t\right)=\partial_{r}F\left(0,t\right)=\partial_{r}P\left(0,t\right)=0,$$

*R* (*r*, 0) = *σ_R_* · *D_N_* (*r*, 0), and *M* (*r*, 0) = *F* (*r*, 0) = *P* (*r*, 0) = 0.

Our chemical equations are similar to those in Graham et al. (2012). A very significant change is that the system is triggered by ROS released by cells as they become necrotic. The initial condition of ROS is not zero. We use the diffusion and natural decay rates of chemicals as estimated in Wang et al. (2014).

The ECM is assumed to be degraded by inflammatory cytokines such as IL-6, measured in terms of decreased proteoglycan concentration in the matrix. When ECM is intact, the sulfate groups are kept inside the ECM. The release of sulfate groups is an indication of ECM degradation, which can be estimated by the decrease in concentration of SO_4_. The average concentration of SO_4_ in normal undamaged cartilage is 30 g/L (Farndale et al., 1982), which is the initial density of ECM in this model. EPO concentration also affects ECM degradation.

We define the Heaviside function,

(2) $$H\left(\theta \right)=\left\{\begin{array}{cc} 1,\; & \theta >0,\\ 0,\; & \theta <1. \end{array}\right.$$

The equation for the ECM dynamics is

(3) $$\partial_{t}\underset{\mathrm{ECM}}{\underset{︸}{U\left(r,t\right)}}=\underset{\mathrm{degradated}\;\mathrm{by}\;\mathrm{alarmins},\;\mathrm{checked}\;\mathrm{by}\;\mathrm{EPO}}{\underset{︸}{-\delta_{U}U\frac{F}{\lambda_{F}+F}H\left(P_{c}-P\right)}},$$

with initial condition *U*(*r*, 0) = 30 mg. The Heaviside function *H*(*P_c_* − *P*) represents the property that ECM degradation can be terminated when the level of *P* exceed *P_c_*.

A substantial difference between our model and Graham et al. (2012) is that we use age structure to represent the time delays for cells to become EPO producing and EPOR-active, instead of delay terms. This gives us a system that is more tractable computationally and more flexible in representing the transitions. Another major difference is that we capture more fully the dynamics among healthy cells. We separate healthy cells into three states, *C_U_*, *C_T_*, and *C_E_*. Since there are only two transitions, *C_T_* → *C_E_* and *S_T_* → *S_A_*, that involve a time delay, we include age structure *a* in only the equations for *C_T_* and *S_T_*. Following Ayati (2007a), we capture the sharp age of transition using the function

(4) $$\gamma \left(a-a_{\max}\right)=\frac{\gamma_{0}}{\sigma }\;\left(\tanh\;\left(\frac{a-a_{\max}}{\sigma }\right)+1\right),$$

where σ is the spread parameter and γ_0_ is the height parameter. For a fixed γ_0_ and σ → 0, cells *C_T_* and *S_T_* switch their states to *C_E_* and *S_A_* all at the same age *a*_max_. When σ is small but not zero, this behavior is smoothed out, better representing the underlying biology. For definiteness, we choose *a*_max_ = 1 for *C_T_* → *C_E_* and $a_{\max}=\frac{1}{2}$ for *S_T_* → *S_A_*.

The equation for the population density of healthy cells not yet signaled by ROS is

(5a) $$\partial_{t}C_{U}\left(r,t\right)=\underset{S_{A}\overset{\mathrm{EPO}}{\underset{\;}{\to }}C_{U}}{\underset{︸}{\alpha_{1}S_{A}\frac{P}{\lambda_{P}+P}}}+\underset{C_{E}\overset{\mathrm{EPO}}{\underset{\;}{\to }}C_{U}}{\underset{︸}{\alpha_{2}H\left(P-P_{c}\right)C_{E}}}-\underset{C_{U}\overset{\mathrm{ROS}}{\underset{\;}{\to }}C_{T}}{\underset{︸}{\beta_{13}C_{U}\frac{R}{\lambda_{R}+R}}},$$

with initial condition

(5b) $$C_{U}\left(r,0\right)=\left\{\begin{array}{cc} 0,\; & 0\leq r\leq 0.25\mathrm{cm}\\ 100000\;\mathrm{cells}∕cm^{2},\; & \mathrm{otherwise}. \end{array}\right.$$

The equation for the population density of healthy cells signaled by ROS and in the process of becoming catabolic is

(6a) $$\begin{array}{llll} & \partial_{t}C_{T}\left(r,a,t\right)+\partial_{a}C_{T}\left(r,a,t\right)=\; & & \;\\ & \;-\underset{C_{T}\overset{\mathrm{DAMPs}}{\underset{\;}{\to }}S_{T}}{\underset{︸}{\beta_{11}\frac{M}{\lambda_{M}+M}H\left(P_{c}-P\right)C_{T}\left(r,a,t\right)}}-\underset{C_{T}\overset{\mathrm{TNF}-\alpha }{\underset{\;}{\to }}S_{T}}{\underset{︸}{\beta_{12}\frac{F}{\lambda_{F}+F}H\left(P_{c}-P\right)C_{T}\left(r,a,t\right)}}\; & & \;\\ & \;-\underset{C_{T}\overset{\mathrm{ROS}}{\underset{\;}{\to }}C_{E}}{\underset{︸}{\kappa_{2}\gamma \left(a-\tau_{2}\right)\frac{R}{\lambda_{R}+R}C_{T}\left(r,a,t\right)}}, \end{array}$$

with age boundary condition

(6b) $$C_{T}\left(r,0,t\right)=\underset{C_{U}\overset{\mathrm{ROS}}{\underset{\;}{\to }}C_{T}}{\underset{︸}{\beta_{13}C_{U}\frac{R}{\lambda_{R}+R}}},$$

and initial condition *C_T_* (*r*, *a*, 0) = 0.

The equation for the population density of healthy cells signaled by ROS and producing EPO is

(7) $$\begin{array}{ll} \partial_{t}C_{E}\left(r,t\right) & =\underset{C_{T}\overset{\tau_{2}\;\mathrm{delay}}{\underset{\;}{\to }}C_{E}}{\underset{︸}{\int_{0}^{\infty }\;\kappa_{2}\gamma \left(a-\tau_{2}\right)\frac{R\left(r,t\right)}{\lambda_{R}+R\left(r,t\right)}C_{T}\left(r,a,t\right)\mathrm{da}}}-\underset{C_{E}\overset{\mathrm{EPO}}{\underset{\;}{\to }}C_{U}}{\underset{︸}{\alpha_{2}H\left(P-P_{c}\right)C_{E}}}, \end{array}$$

with initial condition *C_E_* (*r*, 0) = 0.

The equations for the population density of sick cells in the catabolic state is

(8a) $$\begin{array}{ll} \partial_{t}S_{T}\left(r,a,t\right)+\partial_{a}S_{T}\left(r,a,t\right) & =-\underset{S_{T}\overset{\mathrm{TNF}-\alpha ,\;\mathrm{DAMPs}}{\underset{\;}{\to }}D_{A}}{\underset{︸}{\mu_{\mathrm{ST}}\frac{F}{\lambda_{F}+F}\frac{M}{\lambda_{M}+M}S_{T}\left(r,a,t\right)}}-\underset{S_{T}\overset{\mathrm{TNF}-\alpha }{\underset{\;}{\to }}S_{A}}{\underset{︸}{\eta \cdot \gamma \left(a-\tau_{1}\right)\frac{F}{\lambda_{F}+F}S_{T}\left(r,a,t\right)}}, \end{array}$$

with age boundary condition

(8b) ST(r, 0, t)=∫0∞(β11CUMλM+MH(Pc−P)︸CT→DAMPsST+β12CUFλF+FH(Pc−P)︸CT→TNF−αST)CT(r,a,t)da,

and initial condition *S_T_* (*r*, *a*, 0) = 0.

The equation for the population density of EPOR-active sick cells is

(9) $$\begin{array}{l} \partial_{t}S_{A}\left(r,t\right)=\underset{S_{T}\overset{\mathrm{TNF}-\alpha }{\underset{\;}{\to }}S_{A}}{\underset{︸}{\int_{0}^{\infty }\;\eta \cdot \gamma \left(a-\tau_{1}\right)\frac{F\left(r,t\right)}{\lambda_{F}+F\left(r,t\right)}S_{T}\left(r,a,t\right)\mathrm{da}}}-\underset{S_{A}\overset{\mathrm{EPO}}{\underset{\;}{\to }}C_{U}}{\underset{︸}{\alpha_{1}S_{A}\frac{P}{\lambda_{P}+P}}}-\underset{S_{A}\overset{\mathrm{TNF}-\alpha }{\underset{\;}{\to }}D_{A}}{\underset{︸}{\mu_{S_{A}}\frac{F}{\lambda_{F}+F}S_{A}}}, \end{array}$$

with initial condition *S_A_* (*r*, 0) = 0.

The equation for the necrotic cell population is

(10a) $$\partial_{t}D_{N}\left(r,t\right)=-\underset{\mathrm{natural}\;\mathrm{decay}}{\underset{︸}{\mu_{D_{N}}D_{N}}},$$

with initial condition

(10b) $$D_{N}\left(r,0\right)=\left\{\begin{array}{cc} 100000\;\mathrm{cells}∕cm^{2},\; & 0\leq r\leq 0.25\mathrm{cm},\\ 0,\; & \mathrm{otherwise}. \end{array}\right.$$

#### 2.1.3 Numerical methods

The computational methods used to solve the age- and space-structured differential equations were developed and analyzed in Ayati (2000, 2007a) and Ayati and Dupont (2002, 2009) and have been used effectively and efficiently in the modeling and simulation of biofilms (Ayati and Klapper, 2007, 2012; Klapper et al., 2007; Ayati, 2011), avascular tumor invasion (Ayati et al., 2006), and *Proteus mirabilis* swarm colony development (Ayati, 2006, 2007b, 2009). Numerical efficiency is the main reason that we use age structure rather than delay terms in the model in this paper. To solve the model equations in Graham et al. (2012) and Wang et al. (2014), the authors first did a semi-discretization in space and then solved the resulting system of ordinary delay differential equations using the Matlab function dde23 (Shampine and Thompson, 2001). The function dde23 uses an explicit time integration method. As a result, the stability constraints of the semi-discretized system result in very small time steps. Although another numerical method for delay differential equations could be constructed without such constraints, the need to store the past history of a large spatial system renders the delay approach much less efficient than using age structure; the extra dimension of age is accurately discretized with far less data storage and computation (we use 129 age intervals in the simulations in this paper). The time discretization is adaptive (Ayati and Dupont, 2005) and controlled by a tolerance parameters. We use center finite differences to discretize the diffusion terms.

The relative errors obtained in our computational convergence studies of the numerical results are given in Table 1. For simplicity, we vary the number of spatial intervals. We see that even for a fairly coarse discretization using 100 intervals, we have relative errors of <0.3%.

**Table 1:** **Table of relative errors**.

Variable number of age intervals	100	200	400	800
Healthy normal (*C_U_*)	4.1060E-04	2.2420E-05	2.8220E-06	9.5317E-07
Healthy pre-catabolic (*C_T_*)	1.0342E-03	1.1883E-04	8.5328E-05	1.0001E-04
Healthy EPO producing (*C_E_*)	1.4318E-03	7.7614E-05	1.0574E-05	3.1481E-06
Catabolic (*S_T_*)	1.6966E-03	1.8716E-04	4.1704E-05	6.8092E-05
EPOR-active (*S_A_*)	1.7993E-03	1.0926E-04	1.2838E-05	4.6493E-06
ECM (*M*)	5.5031E-07	6.9499E-08	2.8295E-08	1.5726E-08
IL-6 (*F*)	8.5298E-04	7.1301E-05	1.5445E-05	5.9705E-06
EPO (*P*)	1.8318E-03	1.1066E-04	1.9243E-05	4.6840E-06
DAMPs (*M*)	1.8736E-03	8.5046E-05	2.8458E-05	6.3479E-06
ROS (*R*)	2.1849E-03	5.2076E-04	1.2122E-04	3.0536E-05

*For each dependent variable in the model, we show the relative 2-norm error over all spatial and temporal nodes. Relative errors are calculated by comparing the solution using *n* spatial intervals with the solution using 2*n* spatial intervals. Even for a fairly coarse discretization using 100 intervals, we have relative errors of < 0.3%*.

### 2.2 Experimental material and methods

#### 2.2.1 Generation of cartilage specimens

Osteochondral explants were surgically excised from bovine lateral tibial plateaus (25 mm × 25 mm × 10 mm). All specimens were allowed to equilibrate for two days in culture media in a low oxygen environment (5% O_2_, 5% CO_2_). After equilibration (day 0), explants either underwent a sham impact procedure or were impacted with a 5.5 mm rounded edge indenter from a drop tower, imparting an energy of 2.18 J/cm^2^. Unimpacted explants were removed on day 0, preserved and embedded in paraffin. Impacted explants were placed back in fresh culture media, and removed at day 1, 7, or 14 for preservation and paraffin embedding. Culture media was changed every two days for explants cultured out to day 7 or 14.

#### 2.2.2 Immunohistochemistry

Paraffin embedded cartilage explants were cut into 5 μm thick sections on glass slides to be processed for immunohistochemical detection of interleukin-6 (IL-6), erythropoietin (EPO), or the erythropoietin receptor (EPOR). All cartilage sections for each stain were processed in batch at the same time under identical conditions. The sections were deparaffinized, quenched of endogenous peroxidase activity, and then underwent antigen retrieval using a 0.01 M citric acid solution for 4 h at 65°C. Non-specific antigen binding was then blocked for 1 h with PBS containing 10% goat serum, 1% bovine serum albumin (w/v), and 0.1% tween 20. This blocking solution was then used to dilute the primary antibodies against IL-6 (Abcam ab193799), EPO (Abcam ab65394), and the EPOR (Abcam ab83696) at a ratio of 1:100 from their starting concentration. The solution containing the primary antibody was incubated on the slides overnight at 4°C. The slides were then rinsed and blocking solution applied for 30 min at room temperature. A biotinylated secondary antibody against the primary antibody was then incubated on the slides at a 1:250 dilution (Vector Labs). The slides were then rinsed and incubated with the VECTASTAIN^®^ ABC Reagent (Vector Labs) for 30 min at room temperature. After another rinsing, DAB (3, 3-diaminobenzidine) HRP substrate solution (Vector Labs DAB substrate kit) was incubated on the sections for 2–10 min (depending on primary antibody). The sections were then rinsed for a final time, counter stained with eosin, dehydrated, and a glass coverslip was attached and sealed with permount (Fisher).

Slides were then digitally scanned on an Olympus VS110. The Olympus software controlled the slide stage in all axes and automatically focused/captured high resolution images (322 nm/pixel) which were then tiled together, resulting in full explant immunohistochemically labeled images. The resolution of the resulting images was then reduced to 20% of their original size and exported as tiff images for analysis.

**Figure 2:**
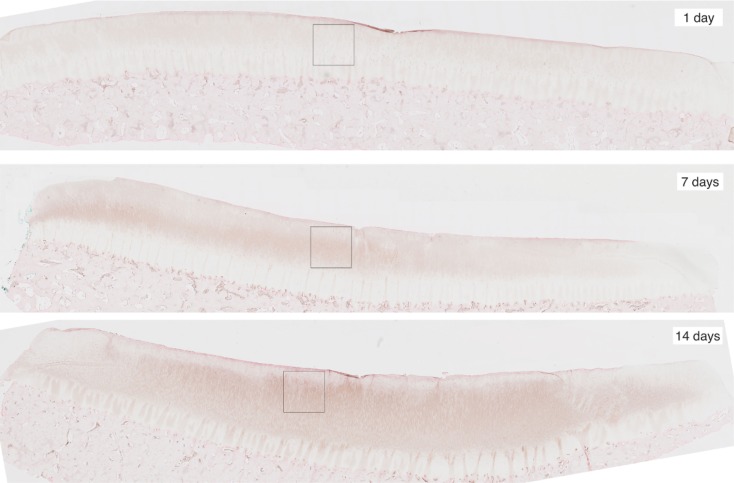
**Slices of articular cartilage created at 1, 7, and 14 days after a single blunt impact**. The impact site appears as a dent at the top of each image. The squares in each region are the zoomed areas shown in Figure 3. These slices have been stained to measure EPO concentrations, which are shown to increase as time progresses.

To obtain the validation data, we analyzed 12 cartilage slices. The slices were separated into six categories with two slices per category: EPO at days 1, 7, 14; and IL-6 at days 1, 7, 14. The length of each slice is around 1″ (2.5 cm approximately). The sham impact was placed in the middle of each cartilage slice and formed a dent (see Figure 2). Cells inside the cartilage slices were stained by the antibodies, and the number of cells per area corresponds to the relative concentrations of EPO and IL-6.

For image processing, we split each slice into two half-slices, divided at the point of impact. From these we could obtain four measurements of each of EPO and IL-6 at each radial value. We further subdivided each half-slice into pieces approximately 0.15 cm long; the pieces correspond roughly to the radius intervals [0, 0.15] cm, [0.15, 0.3] cm, [0.3, 0.45] cm, [0.45, 0.6] cm, [0.6, 0.75] cm, and [0.75, 0.9] cm.

**Figure 3:**
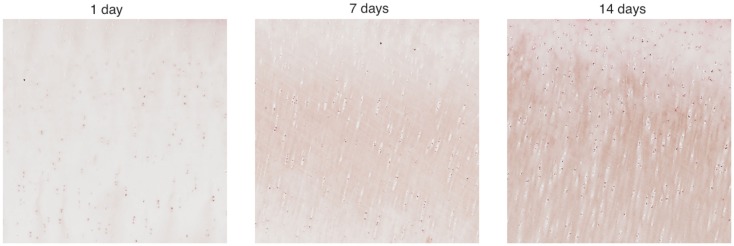
**Zoomed areas from Figure 2**. These samples have been stained to measure EPO concentrations, which are shown to increase as time progresses.

We cropped an 800 × 800 pixel sample image from each piece and estimated the cell numbers in each sample. Figure 3 shows example samples from slices shown in Figure 2. The 800 × 800 pixel sample image has an area of 0.0166 cm^2^. We have four such sample images per radius interval. Average cell count is used as the estimator of cell numbers in each radius interval. The EM algorithm and K-mean methods were applied for the cell segmentation. The upper threshold of the positive cell size is 50–150 μm^2^ (approximately 20–60 pixels), while the lower threshold of the positive cell size is 25–30 μm^2^ (approximately 10–15 pixels).

## 3 Results

### 3.1 Computational results

We simulate the change of the chondrocyte population densities over a 14-day period after an initial cartilage injury in the center of a disk with a radius of 2.5 cm. The radius of the area of impact is 0.25 cm. We assume the initial cells density is 100,000 cells/cm^2^.

The parameters used for the simulations are shown in Table 2. Most of the parameters are the same as in Wang et al. (2014), which contains detailed descriptions of how many of the parameters were determined from the literature. The parameters α_1_, β_11_, β_12_, β_13_, κ_1_, and κ_2_, which determine the cells transition rates, have been changed or added to account for the transition from delay terms to age structure in our model. Because our trigger *P* > *P_c_* is never reached, the value of α_2_ is irrelevant. We include it for completeness of the general model. Similarly, Λ is set sufficiently high that the effect of inflammatory cytokines on the transition to EPO-producing healthy cells (*C_E_*) is neglected; this transition is assumed to be dominated by ROS (*R*) in these simulations.

**Table 2:** **Table of parameters**.

Parameter	Value	Units	Source
*D_R_*	0.1	$\frac{cm^{2}}{\mathrm{day}}$	Graham et al. (2012)
*D_M_*	0.05	$\frac{cm^{2}}{\mathrm{day}}$	Graham et al. (2012)
*D_P_*	0.005	$\frac{cm^{2}}{\mathrm{day}}$	Graham et al. (2012)
*D_F_*	0.05	$\frac{cm^{2}}{\mathrm{day}}$	Graham et al. (2012)
δ*_R_*	60	$\frac{1}{\mathrm{day}}$	Wang et al. (2014)
δ*_M_*	0.5545	$\frac{1}{\mathrm{day}}$	Ito et al. (2008), Wang et al. (2014)
δ*_F_*	0.5545	$\frac{1}{\mathrm{day}}$	Wedlock et al. (1996), Wang et al. (2014)
δ*_P_*	3.326	$\frac{1}{\mathrm{day}}$	Brines and Cerami (2008), Wang et al. (2014)
δ*_U_*	0.0193	$\frac{1}{\mathrm{day}}$	Lu et al. (2011), Wang et al. (2014)
σ*_R_*	0.0024	$\frac{\mathrm{nanomolar}\cdot cm^{2}}{\mathrm{day}\cdot \mathrm{cells}}$	Zhou et al. (2004), Wang et al. (2014)
σ*_M_*	5.17 × 10^−7^	$\frac{\mathrm{nanomolar}\cdot cm^{2}}{\mathrm{day}\cdot \mathrm{cells}}$	Terada et al. (2011), Wang et al. (2014)
σ*_F_*	2.35 × 10^−7^	$\frac{\mathrm{nanomolar}\cdot cm^{2}}{\mathrm{day}\cdot \mathrm{cells}}$	Terada et al. (2011), Wang et al. (2014)
σ*_P_*	4.2 × 10^−5^	$\frac{\mathrm{nanomolar}\cdot cm^{2}}{\mathrm{day}\cdot \mathrm{cells}}$	Brines and Cerami (2008), Wang et al. (2014)
σ*_U_*	0.0154	$\frac{\mathrm{nanomolar}\cdot cm^{2}}{\mathrm{day}\cdot \mathrm{cells}}$	Lu et al. (2011), Wang et al. (2014)
Λ	0.5	nanomolar	Approximated
λ*_R_*	5	nanomolar	Approximated
λ*_M_*	0.5	nanomolar	Approximated
λ*_F_*	0.5	nanomolar	Approximated
λ*_P_*	0.5	nanomolar	Approximated
α_1_	1	$\frac{1}{\mathrm{day}}$	Approximated
α_2_	1	$\frac{1}{\mathrm{day}}$	Approximated
β_11_	100	$\frac{1}{\mathrm{day}}$	Approximated
β_12_	50	$\frac{1}{\mathrm{day}}$	Approximated
β_13_	10	$\frac{1}{\mathrm{day}}$	Approximated
κ_1_	10	$\frac{1}{\mathrm{day}}$	Approximated
κ_2_	10	$\frac{1}{\mathrm{day}}$	Approximated
*P_c_*	1	$\frac{1}{\mathrm{day}}$	Brines and Cerami (2008), Wang et al. (2014)
$\mu_{S_{T}}$	0.5	$\frac{1}{\mathrm{day}}$	Approximated
$\mu_{S_{A}}$	0.1	$\frac{1}{\mathrm{day}}$	Approximated
$\mu_{D_{N}}$	0.05	$\frac{1}{\mathrm{day}}$	Approximated
*τ*_1_	0.5	days	Graham et al. (2012)
*τ*_2_	1	days	Graham et al. (2012)

When we initiate the simulation at *t* = 0, a population of necrotic cells occupies the impact area. We show our results as six series of snapshots in time, taken at *t* = 0, 1, 5, 7, 10, 14 days. We plot days 1, 7, and 14 in particular since those correspond to the attendant experiments. The time length of 14 days is used in our simulation as it is typical of such experimental set-ups.

**Figure 4:**
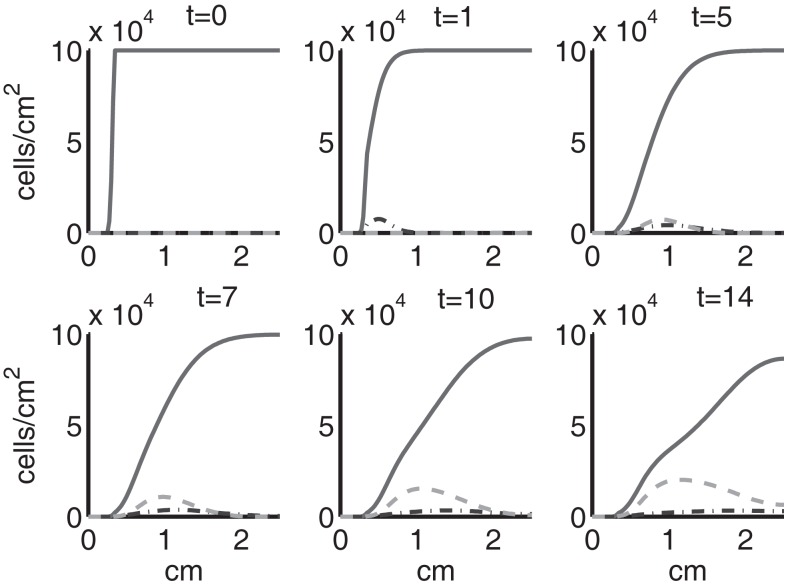
**The densities of healthy cells (*C_U_*, *C_T_*, *C_E_*) at *t* = 0, 1, 5, 7, 10, 14 days**. We show the integral of *C_T_* over all ages to obtain a total population density. *C_U_* is graphed with a solid line, *C_T_* with a dash-dot line, and *C_E_* with a dashed line.

**Figure 5:**
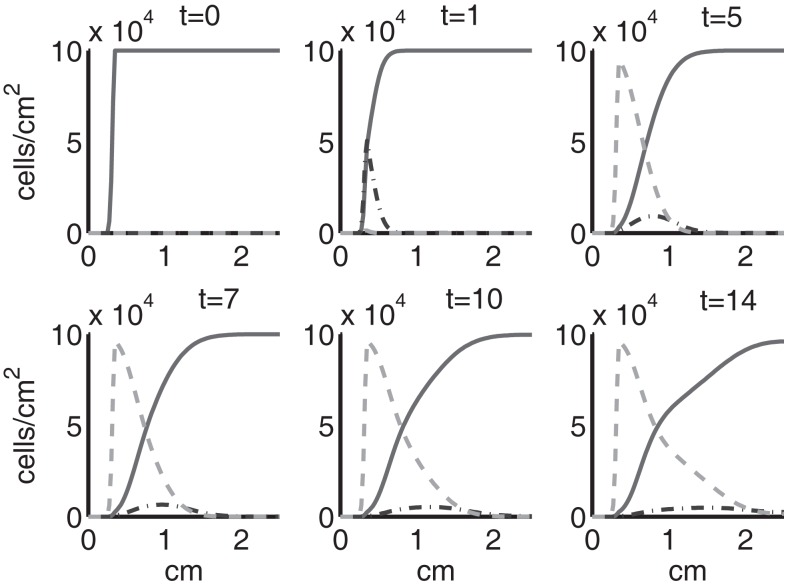
**The densities of healthy, catabolic and EPOR-active cells (*C_U_* + *C_T_* + *C_E_*, *S_T_*, *S_A_*) at *t* = 0, 1, 5, 7, 10, 14 days**. The contribution of pre-catabolic cells is the integral of *C_T_* over all ages to obtain a total population density. We show the integral of *S_T_* over all ages as well to obtain a total population density. Healthy cells are graphed with a solid line, catabolic cells *S_T_* with a dash-dot line, and EPOR-active cells *S_A_* with a dashed line.

**Figure 6:**
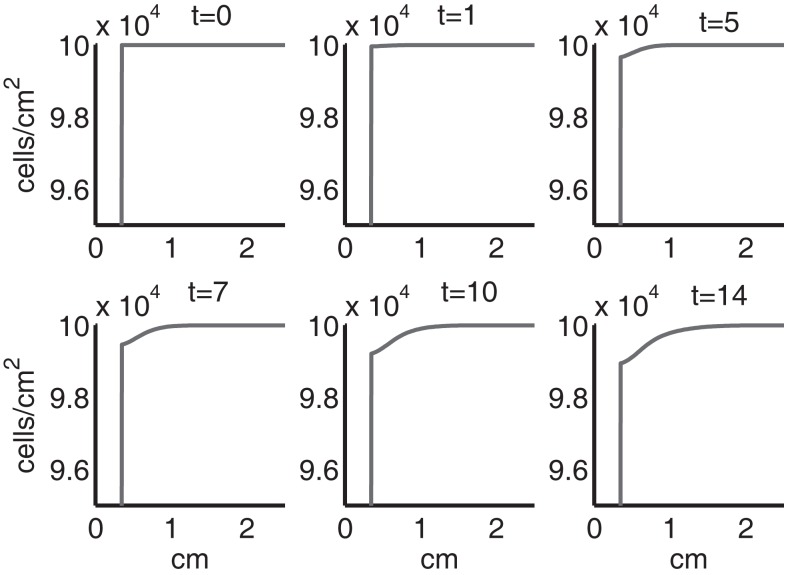
**The density of live cells, (*C_U_* + *C_T_* + *C_E_* + *S_T_* + *S_A_*) at *t* = 0, 1, 5, 7, 10, 14 days**. We show the integrals of *C_T_* and *S_T_* over all ages to obtain total population densities.

**Figure 7:**
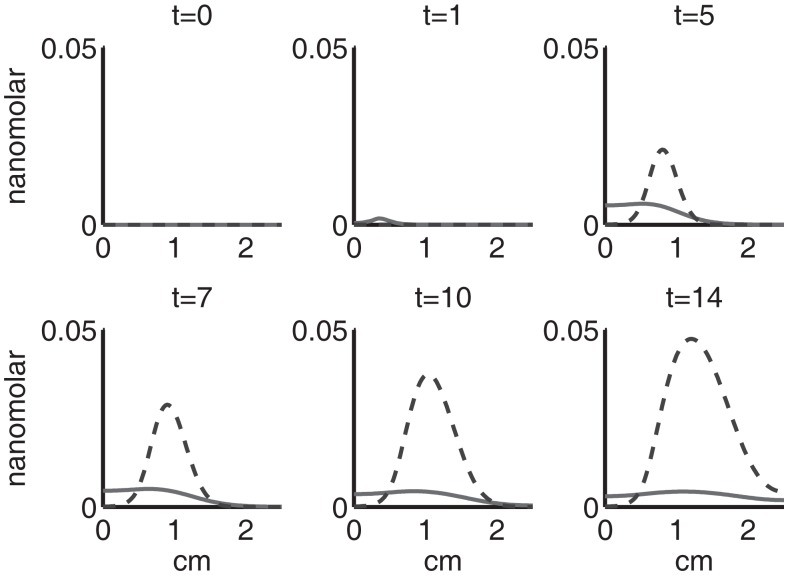
**The concentrations of IL-6 (*F*) and EPO (*P*) at *t* = 0, 1, 5, 7, 10, 14 days**. IL-6 is graphed using a solid line and EPO is graphed using a dashed line.

**Figure 8:**
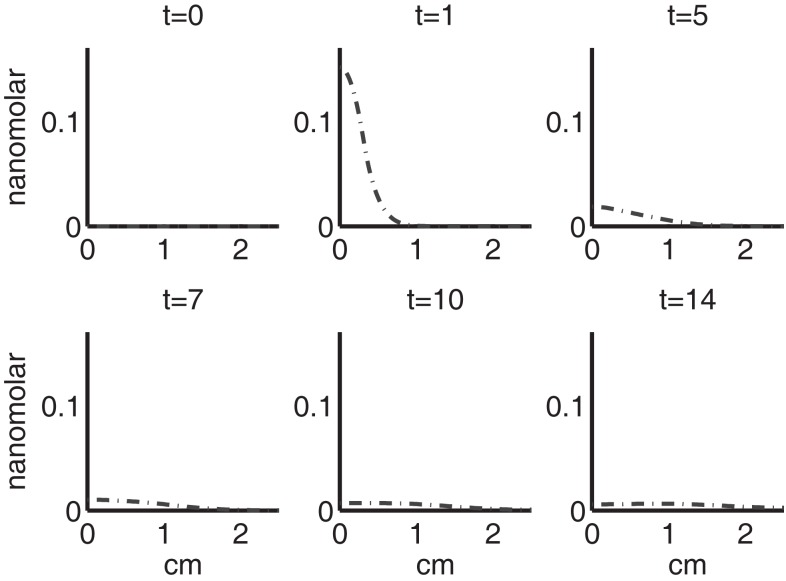
**The concentration of DAMPs (*M*) at *t* = 0, 1, 5, 7, 10, 14 days**.

**Figure 9:**
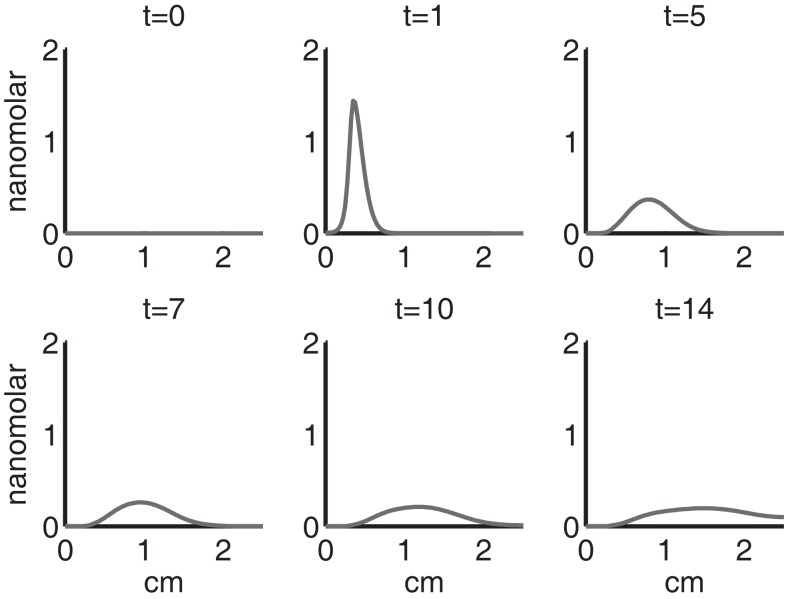
**The concentration of ROS (*R*) at *t* = 0, 1, 5, 7, 10, 14 days**.

Figure 4 shows the population densities of the three states of healthy cells. Figure 5 shows the healthy, catabolic, and EPOR-active cell population densities. Figure 6 shows the live cell population densities, i.e., the populations of all healthy and sick cell classes. Figure 7 compares the concentrations of IL-6 (our representative inflammatory cytokine) and EPO (our primary anti-inflammatory cytokine). Our focus is on the balance of pro- and anti-inflammatory cytokines. We choose IL-6 as representative since it a known inflammatory cytokine. We remark that TNF-α is also a viable choice. Figure 8 shows the concentration of DAMPs. Figure 9 shows the concentration of ROS.

Simulation results show that after the initial injury, the cells adjacent to the impact area quickly sense the release of ROS and begin to switch states. At the same time, DAMPs released by necrotic cells start to trigger the pre-catabolic cell population (*C_T_*) to become catabolic (*S_T_*). These catabolic cells then start to produce more DAMPs, inflammatory cytokines, and ROS that aggravate the inflammation. Because of the non-zero transition times in the *C_T_* → *C_E_* and *S_T_* → *S_A_* transitions, cells cannot produce the anti-inflammatory cytokine EPO nor express a receptor for EPO immediately. The earliest an anti-inflammatory response can occur is day 1 (shown in the simulation figures). In Figures 4–6, we see that the population density of healthy cells *C_U_* starts to decline as those cells switch to the pre-catabolic and catabolic states (*C_T_* and *S_T_*). By day 5, we observe a significant density of pre-catabolic (*C_T_*), EPO producing (*C_E_*) and catabolic (*S_T_*) cells. From day 5 onward, there is some cell death adjacent to the impact area followed by the continuing spread of the cartilage lesion. Due to the low release rate of EPO by the *C_E_* population, we do neither have significant conversion of *C_E_* cells to *C_U_* cells nor do EPOR-active cells (*S_A_*) recover to a healthy state. By day 10, there is an appreciable density of EPO-producing cells (*C_E_*), and EPOR-active cells (*S_A_*) awaiting EPO to finish their conversion to the healthy state (*C_U_*). However, the proportion of sick cells to healthy cells continues to grow during the simulation period, which was chosen to match common experimental set-ups.

In the simulation period of 14 days, the decrease of ECM density is quite small. This is because ECM degradation is a much slower process than the apoptosis of cells.

Figures 7–9 show the concentration of the chemical concentrations: IL-6, EPO, DAMPs, and ROS. The EPO concentration is too low to trigger the anti-inflammatory process efficiently, which is an expected result (Brines and Cerami, 2008). Since cells will release a small amount of ROS before they become necrotic, the concentration of ROS is not zero inside the impact area at *t* = 0. Although the initial burst of ROS decreases rapidly due to the fast decay rate of ROS, the production of ROS is continued by catabolic cells *S_T_*. This results in a lower but broader concentration of ROS, as expected (Brouillette et al., 2013). DAMPs concentrations peak are early at the site of the impact.

The main competition between pro- and anti-inflammatory cytokines results in a steady increase of IL-6 across the cartilage, whereas EPO is more heavily concentrated just outside the penumbra of the inflamed region. The net result is a slowing, but not full cessation, of the spread of the inflammation.

We remark that in the simulations presented, we never reach the threshold *P* > *P_c_*. This is consistent with (Brines and Cerami, 2008) that chondrocytes alone cannot produce enough EPO to stop the cartilage inflammation, so that *H*(*P_c_* − *P*) = 1 and *H*(*P* − *P_c_*) = 0 for all *r* and *t*. Brines and Cerami (2008) described the possibility of EPO therapy for cartilage injury, in which case *P* may exceed *P_c_*. We note that the model we have formulated is set up to be modified for such therapies.

#### 3.1.1 Parameter sensitivity analysis

We examined the approximated parameters in Table 2 for sensitivity, with the exception of α_2_ and Λ, which do not affect the system in these simulations, but are included in the model for generality. Holding all other parameters to their base value in Table 2, we perturbed a given parameter. We found a region around each parameter base value where the qualitative behavior of the system did not change. The perturbed values for each parameter and an interval in which the system did not change qualitatively or appreciably quantitatively are shown in Table 3. Specifically, as each parameter was increased within its perturbation range, we found most noticeably that the following densities and concentrations decrease:

- for λ*_R_*, EPOR-active cells (*S_A_*) and DAMPs (*M*);
- for λ*_M_*, sick cells (*S_T_*, *S_A_*), IL-6 (*F*), ROS (*R*), and DAMPs (*M*);
- for λ*_F_*, EPOR-active cells (*S_A_*) and DAMPs (*M*);
- for λ*_P_*, normal healthy cells (*C_U_*), pre-catabolic healthy cells (*C_T_*), catabolic cells (*S_T_*), IL-6 (*F*), EPO (*P*), and DAMPs (*M*);
- for α_1_, all variables, except for EPOR-active cells (*S_A_*) and ECM (*U*);
- for β_11_, all healthy cells (*C_U_*, *C_T_*, *C_E_*), ECM (*U*), and EPO (*P*);
- for β_12_, all healthy cells (*C_U_*, *C_T_*, *C_E_*) and EPO (*P*);
- for β_13_, normal healthy cells (*C_U_*) and ECM (*U*);
- for κ_1_, healthy pre-catabolic cells (*C_T_*), catabolic cells (*S_T_*), EPOR-active cells (*S_A_*), IL-6 (*F*), ROS (*R*), and DAMPs (*M*);
- for κ_2_, healthy pre-catabolic cells (*C_T_*), EPOR-active cells (*S_A_*), IL-6 (*F*), ROS (*R*), and DAMPs (*M*);
- for $\mu_{S_{T}}$, none (we see no appreciable change for the interval around the base parameter);
- for $\mu_{S_{A}}$, none (we see no appreciable change for the interval around the base parameter);
- for $\mu_{D_{N}}$, normal healthy cells (*C_U_*), pre-catabolic healthy cells (*C_T_*), and all chemicals (*R*, *M*, *F*, *P*);

**Table 3:** **Table of perturbed parameter values**.

Parameter	Base value	Perturbed values	Interval
λ*_R_*	5	{1, 3, 7, 9}	[3, 7]
λ*_M_*	0.5	{0.1, 0.3, 0.7, 0.9}	[0.3, 0.7]
λ*_F_*	0.5	{0.1, 0.3, 0.7, 0.9}	[0.3, 0.7]
λ*_P_*	0.5	{0.1, 0.3, 0.7, 0.9}	[0.3, 0.9]
α_1_	1	{0.1, 0.5, 1.5, 2}	[0.5, 2]
β_11_	100	{50, 75, 125, 150}	[60, 70]
β_12_	50	{40, 45, 55, 60}	[40, 60]
β_13_	10	{1, 5, 15, 20}	[5, 15]
κ_1_	10	{1, 5, 15, 20}	[5, 20]
κ_2_	10	{1, 5, 15, 20}	[5, 20]
$\mu_{S_{T}}$	0.5	{0.1, 0.3, 0.7, 0.9}	[0.1, 0.9]
$\mu_{S_{A}}$	0.1	{0.01, 0.05, 0.15, 0.2}	[0.01, 0.2]
$\mu_{D_{N}}$	0.05	{0.01, 0.03, 0.07, 0.09}	[0.01, 0.09]

*For each of the approximated parameter values in the model, we show the perturbed values in which we conducted additional simulations, and present an interval in which we found no meaningful qualitative or quantitative change from the simulation conducted with the corresponding base parameter given in Table 2*.

The most sensitive parameters in the system are λ*_R_*, λ*_M_*, and λ*_F_*. In particular, when these parameters are relatively small compared to their base value, solutions that are otherwise monotone become non-monotone or somewhat oscillatory.

### 3.2 Experimental validation

**Figure 10:**
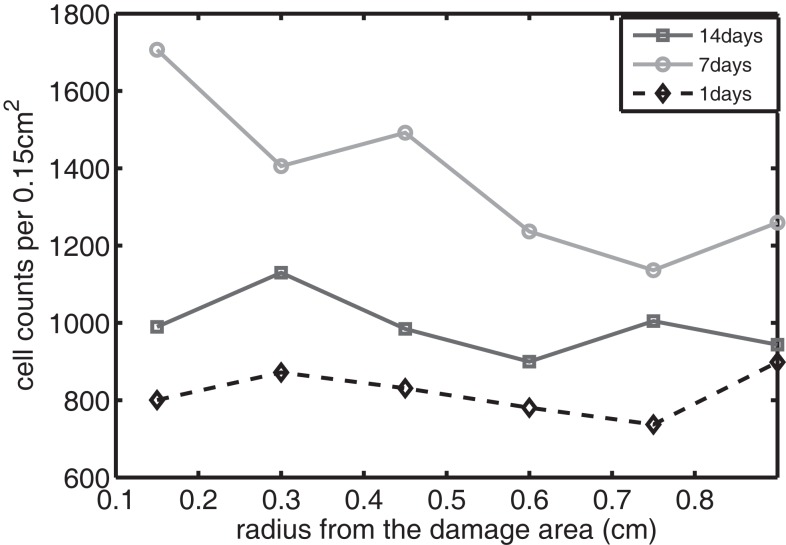
**The concentration of IL-6 measured experimentally**. Cell counts per 0.15 cm^2^ is the density of chondrocytes expressing IL-6 at a level detectable by immunohistochemistry.

**Figure 11:**
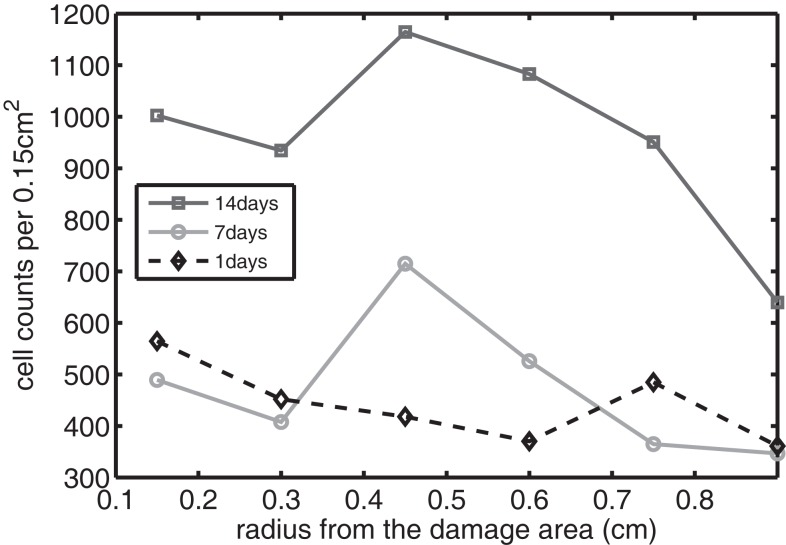
**The concentration of EPO measured experimentally**. Cell counts per 0.15 cm^2^ is the density of chondrocytes expressing EPO at a level detectable by immunohistochemistry.

The sequence and distributions of the cell states match what is expected from past and current understanding of the “penumbra” around a lesion, in which an initial burst of pro-inflammatory cytokine expression is eventually attenuated by an increase in anti-inflammatory cytokine expression. Our major simulation result predicting this progression (Figure 7) was mainly in agreement with experimental results showing an acute post-injury increase in IL-6 expression and decrease in EPO expression, with both factors gradually returning to baseline at 14 days (Figures 10 and 11, respectively), signaling the end of the injury response cycle. It is noteworthy that our simulation does not predict a runaway pro-inflammatory response. This is also consistent with experimental findings, which showed that the initial damage done to the cartilage matrix by impact does not progress over time. Importantly, these results imply that additional factors beyond the single impact insult are required to drive further cartilage degeneration. Such factors may include synovitis or abnormal joint loading, which accompany most joint injuries.

We bring attention to some specific features of the validation result. Figure 11 shows higher concentrations of EPO in day 14 than earlier. In Figure 10, the concentration of IL-6 in day 7 is higher than the concentration in day 1 or day 14. These correspond to our simulation results in Figure 7.

**Figure 12:**
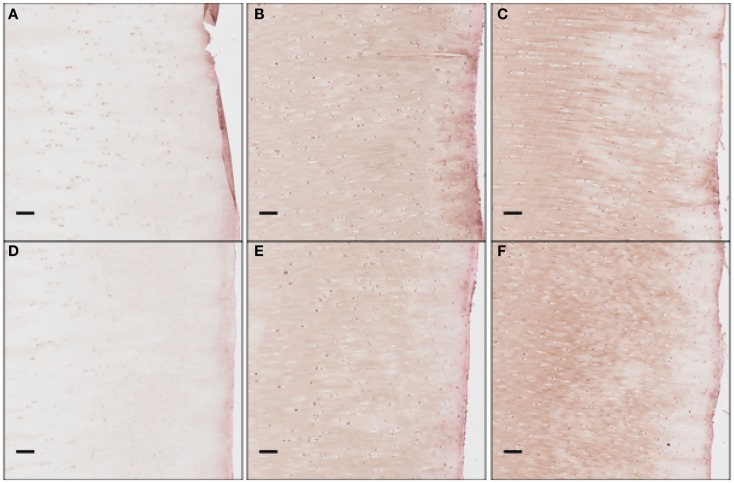
**The concentration of EPO as a function of “positive cells.”** Each scale bar is 100 μ. Immunohistochemistry images were analyzed for “positive cells,” indicating that they were above the background thresholding technique and within our cell size criteria. **(A)** shows concentration at day 1 at the impact site, **(B)** at day 7 at the impact site, **(C)** at day 14 at the impact site, **(D)** at day 1 a distance 2 mm from the impact site, **(E)** at day 7 at 2 mm away, and **(F)** at day 14 at 2 mm away. We see clearly EPO being less constitutively expressed at day 1, when compared to day 7 or 14.

We clarify that our current experimental techniques cannot extract precise concentration values for cytokines throughout the cartilage matrix. Instead, they measure the numbers of chondrocytes expressing each cytokine at a level detectable by immunohistochemistry (“positive cells”). Figure 12 shows these proteins being expressed and then released into extracellular space. Although there are limitations to counting only positive cells in this fashion, for validation purposes of this model this type of analysis is more than sufficient.

The immunohistochemistry analysis indicates relative rises and declines in the amounts of cytokine present in and around individual cells where they may act in autocrine or paracrine fashion. Thus, the immunohistochemical approach gives a rough estimation of the distribution of bioavailable cytokines. In this sense, we see agreement with our predictions: the broad, nearly uniform, distributions of IL-6, peaking near day 7; and the more unimodal distributions of EPO, increasing steadily.

## 4 Discussion

We present an age- and space-structured model to simulate the development of an articular cartilage lesion after a single blunt impact. We simplified the model to a radially symmetric geometry. Based on previous experimental findings, we hypothesized that the consequences of mechanical trauma to cartilage depend on the balance between competing chondrolytic and chondroprotective responses of local chondrocytes. Whether damaged cartilage is stabilized, or begins down the path to progressive degeneration, has been regarded as a matter of great biologic complexity. However, our mathematical simulation confirms that the complex behavior of this system can be modeled using just two cytokines with opposing pro- and anti-inflammatory activities. The post-injury pattern of expression for IL-6 and EPO indicated by immunohistology suggests they may play roles in this binary system.

Our model did not predict runaway degeneration after a simulated impact injury. This result is appropriate and matches bench experiments showing that explanted cartilage recovers from impacts of similar magnitude, which cause minimal structural damage. Structurally damaging impacts deserve attention, but will require consideration of spatial heterogeneity in the impact site, which is beyond the scope of the current work. In addition, extrinsic factors that are sure to influence cartilage recovery *in vivo*, including mechanical stress and synovitis, must eventually be incorporated in the simulation to predict *in vivo* outcomes. Adding synovitis to our model as a second source of pro-inflammatory/pro-chondrolytic cytokines is computationally straightforward, but awaits parameterization data from *in vivo* models. Adding mechanical stress effects involves a much more extensive effort to combine our biomathematical model with a finite element model, which is the subject of ongoing research.

In summary, although our simulation results were obtained using a relatively small number of approximated parameters, our calculations provide useful predictions of the formation of cartilage lesions after a single blunt impact. Although we limited simulations to only 2 weeks in this work, it is possible to predict outcomes over the much longer time frames. Thus, our *in silico approach* may ultimately enable us to examine the long-term effects of various joint injury scenarios in people, which is difficult or impossible to address in experimental models.

## Author Contributions

XW and BA developed the mathematical model and software used to solve the model equations. XW conducted the numerical simulations. MB conducted the immunohistochemistry, and slide preparation and scanning for the validation experiments. MB and JM designed the validation experiments. XW, MB, BA, and JM contributed to the writing and editing of the manuscript.

## Conflict of Interest Statement

The authors declare that the research was conducted in the absence of any commercial or financial relationships that could be construed as a potential conflict of interest.
