# Corrigendum: “A Validated Model of the Pro- and Anti-Inflammatory Cytokine Balancing Act in Articular Cartilage Lesion Formation”

**DOI:** 10.3389/fbioe.2015.00073

**Published:** 2015-05-26

**Authors:** Xiayi Wang, Marc J. Brouillette, Bruce P. Ayati, James A. Martin

**Affiliations:** ^1^Program in Applied Mathematical and Computational Sciences, University of Iowa, Iowa City, IA, USA; ^2^Department of Orthopaedics and Rehabilitation, University of Iowa, Iowa City, IA, USA; ^3^Department of Biomedical Engineering, University of Iowa, Iowa City, IA, USA; ^4^Department of Mathematics, University of Iowa, Iowa City, IA, USA

**Keywords:** articular cartilage, structured model, lesion formation and abatement, EPO, IL-6

There are a number of errors in the original manuscript (Wang et al., 2015) due to the use of incorrect versions of our material during the final manuscript preparation stage. They are

1. Figure 1 has the incorrect schematic. The figure in this corrigendum is the schematic that matches the models and simulations in the manuscript.
2. In equation (1b), δ*_U_* should be σ*_U_*.
3. The correct initial condition for ROS is *R*(*r*, 0) = 0.
4. The Heaviside function is defined incorrectly in equation (2). We use the standard Heaviside function, *H*(θ) = 1 for θ ≥ 0, *H*(θ) = 0 for θ < 0.
5. In equations (5a) and (6b), the Monod functions that depend on ROS, *R*, should instead depend on DAMPs, *M*.
6. The parameter *η* in equations (8a) and (9) should be *κ*_1_.
7. The underbraces in equations (8a), (8b), and (9) that indicate dependence on TNF-*α* specifically should instead indicate dependence on a generic pro-inflammatory cytokine, denoted by *F*.
8. Equation (8b) does not depend on *C_U_*. In place of *C_U_* in the equation there should instead be implicit multiplication by 1.
9. In Table 2, the correct parameter value for δ*_F_* is 0.1664 and the correct parameter value for δ*_P_* is 0.5545. These were obtained from the derivation in (Wang et al., 2014):
  (1) $$\begin{array}{ll} & \delta_{F}=-\frac{24}{100\text{ hour}}\ln\;\left(\frac{1}{2}\right)=0.1664∕\text{day}, \end{array}$$
  (2) $$\begin{array}{ll} & \delta_{P}=-\frac{24}{30\text{ hour}}\ln\;\left(\frac{1}{2}\right)=0.5545∕\text{day}. \end{array}$$
  Here 100 hours is the approximate half-life of TNF-α (Wedlock et al., 1996; Brines and Cerami, 2008) and 30 hours is the approximate half-life of DAMPs and EPO (Ito et al., 2008). To obtain the half-lives from the source literature, we used the “N-end Rule” (Varshavsky, 1997). The similarities in their half-lives are why we have that δ*_M_* = δ*_P_*. We note that the parameter values in Table 1 of (Wang et al., 2014) are not consistent with the correct values in the body of the text (Wang et al., 2014, pg. 931).

**Figure 1:**
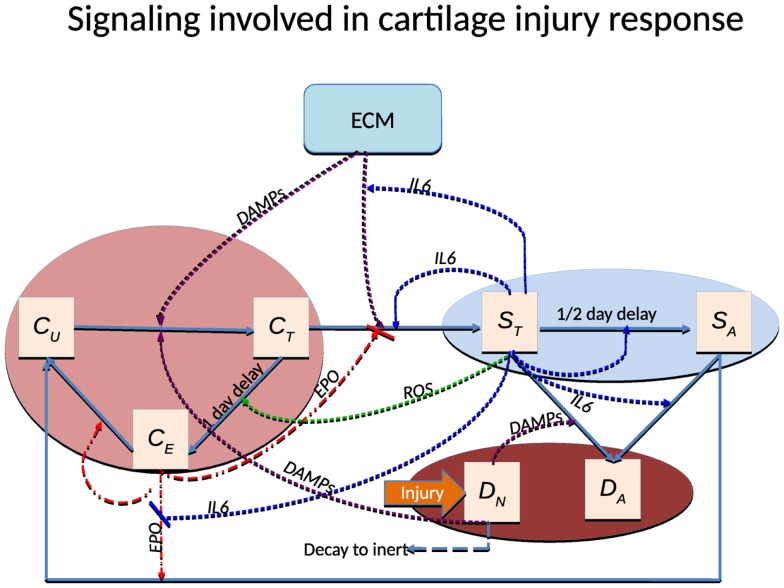
**Schematic of the articular cartilage lesion formation process due to a single blunt impact**.

All the simulations in the original manuscript where conducted with these versions of the equations and parameters.

## Conflict of Interest Statement

The authors declare that the research was conducted in the absence of any commercial or financial relationships that could be construed as a potential conflict of interest.
